# A survey study on student preferences regarding pathology teaching in Germany: a call for curricular modernization

**DOI:** 10.1186/s12909-015-0381-7

**Published:** 2015-06-02

**Authors:** Florian E. M. Herrmann, Markus Lenski, Julius Steffen, Magdalena Kailuweit, Marc Nikolaus, Rajasekaran Koteeswaran, Andreas Sailer, Anna Hanszke, Maximilian Wintergerst, Sissi Dittmer, Doris Mayr, Orsolya Genzel-Boroviczény, Diann S. Eley, Martin R. Fischer

**Affiliations:** 1 Medizinische Fakultät der Ludwig-Maximilians-Universität, Munich, Germany; 2 Molecular and Cellular Pathology, School of Medicine, The University of Queensland, Brisbane, Australia; 3 Pathologisches Institut, Klinikum der Universität München, Munich, Germany; 4 International Student Exchange Office and Neonatologie Innenstadt, Dr. von Haunersches Kinderspital, Klinikum der Universität München, Munich, Germany; 5 School of Medicine, The University of Queensland, Brisbane, Australia; 6 Institut für Didaktik und Ausbildungsforschung in der Medizin, Klinikum der Universität München, Munich, Germany

**Keywords:** Pathology education, Curriculum development, Student survey, Teaching

## Abstract

**Background:**

Pathology is a discipline that provides the basis of the understanding of disease in medicine. The past decades have seen a decline in the emphasis laid on pathology teaching in medical schools and outdated pathology curricula have worsened the situation. Student opinions and thoughts are central to the questions of whether and how such curricula should be modernized.

**Methods:**

A survey was conducted among 1018 German medical students regarding their preferences in pathology teaching modalities and their satisfaction with lecture-based courses. A qualitative analysis was performed comparing a recently modernized pathology curriculum with a traditional lecture-based curriculum. The differences in modalities of teaching used were investigated.

**Results:**

Student satisfaction with the lecture-based curriculum positively correlated with student grades (spearman’s correlation coefficient 0.24). Additionally, students with lower grades supported changing the curriculum (spearman’s correlation coefficient 0.47). The majority supported virtual microscopy, autopsies, seminars and podcasts as preferred didactic methods.

**Conclusions:**

The data supports the implementation of a pathology curriculum where tutorials, autopsies and supplementary computer-based learning tools play important roles.

## Background

While pathology was of central importance in the medical education of physicians in the 20th century and before, it has lost its once held status in the past decades. Increasingly opinions are being voiced that pathology is not important to medical education [1], while there are those who believe that it is central to the understanding of disease [2] and critical to the development of a fine physician, as is our opinion. The negative opinion of pathology teaching held by some hinges on two key factors: the shift in the role of pathology and changes relating to the modern medical student.

Over the years pathology has developed from an autopsy and macroscopy based discipline to a technically finessed histological and molecular field. This change has also been mirrored in the teaching of this field in medical school. While for medical students the importance of the subject should in our opinion lie in the basics of disease (which should be understood) focus has strayed more towards histological and molecular details (which are often merely memorized). Today medical specializations are frequently selected near the beginning of medical school and students work towards a very specific objective. In our experience this has led to an increase in interest for clinical practice in specific medical fields and less for pathology which should actually provide the cornerstone of the students’ knowledge of disease whatever specialization he/she may choose.

Students must understand the importance of pathology as an interdisciplinary field. The knowledge of the physiological normal condition and aetiology of diseases helps to understand pathogenesis which is the basis of all diagnosis and therapy. With pathology teaching hours having decreased over the years [3, 4] this is a difficult aim which requires fine planning. The balancing of teaching hours with the available faculty and student motivation for the field is a major difficulty. For a successful general improvement of the curricula student involvement in the process is essential.

In teaching, students’ wishes regarding the curriculum are of utmost importance. Especially in a field like medicine teaching must be trimmed to assist students in effectively retaining extensive amounts of knowledge. It has been shown that outdated methods, such as lectures with minimal student teacher interaction, are still being used world-wide in pathology teaching [5]. As forerunners in the field, some pathology teaching departments in medical schools were able to integrate modern teaching methods including problem-based learning and computer-based methods (such as virtual microscopy and online cases) into their curricula early on [6]. Due to student dissatisfaction with outdated teaching methods (mainly lecture based) the University of Queensland School of Medicine (SOM) also recently restructured their pathology curriculum.

Hypothesizing that medical students wish for more modern teaching methods in pathology curricula and that student judgment is a valid tool for curriculum development, we set out to survey students of one of Germany’s largest medical faculties (Ludwig Maximilian University, Munich (LMU)) regarding their demands. We aimed to support our hypothesis by comparing the qualitative and quantitative data from this survey with a ‘best practice model’ of pathology teaching found at SOM after its modernization.

## Methods

### Qualitative comparison of a lecture-based with a modernized curriculum

During a 4 month clinical placement at SOM, three German final year medical students took advantage of the recent changes made to SOM’s pathology curriculum by investigating the novel methods used to teach the students. The students participated in courses and performed individual interviews with select faculty. The program’s structure and modalities of teaching were compared with their counterparts at LMU.

### Focus group analysis–a basis for survey construction

Open ended questions regarding the lecture-based pathology curriculum at LMU were developed for interview purposes. Two groups of five students were interviewed for 30 min on topics dealing with pathology didactics and satisfaction with pathology teaching. The participants were informed that all of the recorded data was to be used for curriculum development and scientific study. The interviews were thereafter transcribed to remove the effect of paraverbal cues on further data analysis. Thereafter the interviews were analysed via Mayring’s qualitative context analysis [7]. This involved sequentially processing the transcripts as follows: paraphrasing, investigating constructiveness and generalizability of comments, removing replicates, and bundling similar comments. For the subsequent survey these comments were used as statements and student agreement with these was quantified as to canvas the perceptions of the whole LMU cohort.

### Survey construction

The survey, consisting of 56 question blocks, was previously approved by the ethics committee of LMU. The initial questions investigated student demographics including: gender, age and whether the students had already passed the pathology exam. The actual survey questions were constructed making them available to being answered on a six-level Likert scale (ranging from strongly agree to strongly disagree). All students enrolled in the LMU medical school were invited to participate in the online survey by email. Twenty book vouchers were provided as an incentive for participation; the winners were chosen among all participants by chance.

In the results section data is represented in the following way: the initial percentage value in the brackets signifies cumulative proportion of the students who answered: *strongly agree, agree and slightly agree*, while the second value only represents the proportion that answered *strongly agree*. This representation of the data is meant to show what proportion of the students is in agreement with a statement (any level of agreement–first percentage value) and how high the level of agreement is (highest level of agreement–second percentage value).

### Statistical analysis of survey data

*Statistical Package for Social Sciences* (SPSS for Windows, Version 17.0, SPSS Inc., Chicago, IL, USA) was used for statistical analysis. SPSS and *Microsoft Excel* (Microsoft Excel 2013, Microsoft Corporation, Redmond, WA, USA) were used for data presentation. Subgroups for separate data analysis included students currently participating in pathology courses and students who had finished pathology courses. The mean value and the standard deviation (SD) were determined for each parameter. Student’s *t*-Test for independent variables was used to investigate statistically significant differences between the means of various datasets. The Kolmogorov-Smirnov-Test was used prior to this, to assure normal distribution of the investigated parameters. Correlation between variables was assessed using Spearman’s rho coefficient for ordinal variables. Finally the sub-group of students, which already passed the final pathology exam, was further investigated. A one-way repeated multi-measure ANOVA was used to investigate differences regarding the students’ support for specific methods of teaching. In the graphic representation of data, students who did not answer a question were omitted. A university biostatistics clinic was consulted and confirmed the legitimacy of the described statistical approach.

## Results

### Qualitative comparison of a modern and a traditional lecture-based curriculum

#### The framework of the pathology programs

The current SOM medical degree program is a Bachelor of Medicine Bachelor of Surgery program consisting of 2 foundation years and 2 clinical practice years. Students entering the program have completed a bachelor degree beforehand. The LMU medical degree consists of 2 preclinical and 4 clinical years including an internship. No prior university degree is required to study medicine at LMU. Both SOM’s and LMU’s class sizes are ca. 550 students. Pathology is taught in the first two years at SOM (general pathology in year 1 and special pathology in year 2). At LMU general pathology is taught intensively in year 3, the beginning of the clinical leg, and special pathology is taught alongside the clinical subjects in the following 2 years. SOM consists of a pathology faculty (Head of department 0.3 full-time equivalent and 3 full time senior lecturers) based at SOM supplemented by non-university staff comprising 15 consultants and 40 registrars who are involved in teaching to different extents. LMU’s pathology teaching team consists of eight lecturers. While at SOM there are two faculty members who are solely dedicated to teaching there are no such faculty members at LMU.

#### Differences in modalities of teaching used

At LMU lectures are the main mode of teaching students. In year 3 students attend laboratory courses in which microscopic and macroscopic specimens are viewed. Thereafter pathology is solely taught in clinical pathological conferences.

The major strength of the SOM program is its tutorial which comprises the faculty’s face-to-face time with students. In a group consisting of up to 40 students separated into small groups, macroscopic specimens are circulated in defined intervals while students must solve problems related to their specimens in their group. The lecturer goes through the answers at the end of the session in an interactive manner. Other than the tutorial, SOM also provides filmed lectures to the students via an online portal. Utilizing its expansive pathology museum / integrated pathology learning centre (IPLC) a case of the week is set up on a computer terminal for students to interactively learn using macroscopic specimens.

### Quantification and statistical evaluation of students’ opinions

#### Survey participation

The survey was active for 14 days (in April and May of 2014) during which time 1018 LMU students took part, giving a response rate of 20 %. The mean age of participants was 24.7 years, 56 % of the participants who reported their gender were female, 34 % male, 10 % omitted the question (see Table 1).

**Table 1:** Demographics

Age		
	Years	
Mean	24.69	
Standard deviation	3.39	
Gender		
	Number of students	Percentage
Female	575	56.48
Male	348	34.18
Status of pathology studies		
	Number of students	Percentage
Completed	285	28.00
Not yet completed	359	35.27

Characteristics of the surveyed medical student population. Discrepancies in the number of students and percentages are accounted for by students who did not answer all questions

#### Student goals in pathology

**Fig. 1:**
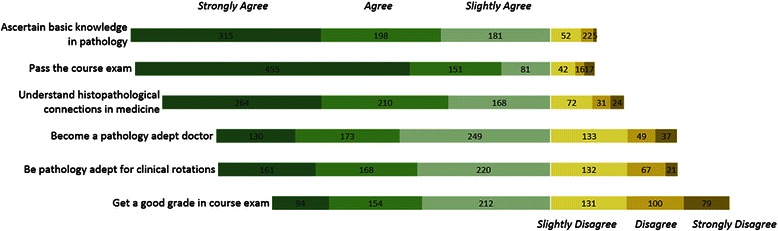
Student goals. Students’ level of agreement with the statement that the labels on the left describe their goals in the pathology course in medical school (absolute values)

Students were asked to evaluate statements regarding their goals in the pathology course. The goals which were found to be most important to students were as follows: pass the course exam (90 % (cumulative agree), 58 % (strongly agree)), ascertaining basic knowledge in pathology (90 %; 40 %) and understanding histopathological connections (83 %, 33 %). For a graphical representation of student goals (in absolute values) see Fig. 1.

#### Student satisfaction and support for change

**Fig. 2:**
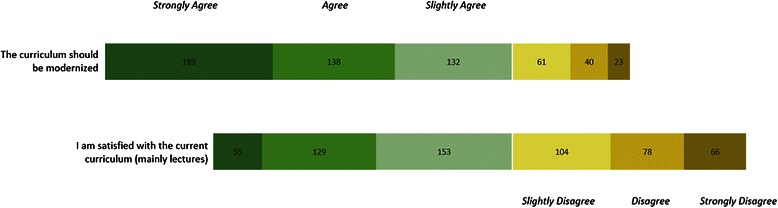
Student satisfaction. Representation of students’ responses to the statement that their pathology curriculum should be modernized and with the statement that they are satisfied with their current curriculum (absolute value). Discrepancy between the two charts suggests that a subgroup of students, while slightly satisfied, would still welcome modernization

When questioned about their satisfaction with the current form of teaching which mainly consisted of lectures only 9 % *strongly agreed* (57 %; 9 %). The proportions that *strongly disagreed*, *disagreed* and *slightly disagreed* added up to 43 %. When asked to evaluate the statement that the current curriculum should be modernized 32 % strongly agreed, 24 % agreed and 23 % slightly agreed (79 %; 32 %). The proportion that selected any answer in the range of disagreement added up to 21 %. These data are represented in Fig. 2.

#### Preferred modalities of teaching

**Fig. 3:**
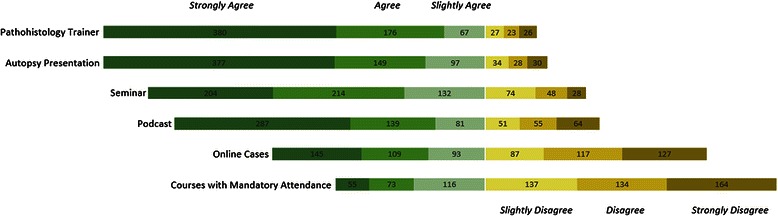
Student preferences. Students’ level of agreement with the statement that the type of course mentioned in the labels on the left should be included in the modernized curriculum (absolute values)

In our survey students were asked whether they agree with the statement that a specific modality of teaching should play a more central role in the pathology curriculum. This question was asked in regards to six different modalities of teaching. “Courses with mandatory attendance” was included although it is in itself not a specific modality of teaching. The students most strongly supported virtual microscopy (89 %; 53 %), autopsy presentation (87 %; 55 %) and seminars (79 %; 32 %) (Munich seminars would constitute a course similar to the tutorial at SOM). Podcasts also received majority support (75 %; 41 %) (see Fig. 3).

The one-way repeated multi-measure ANOVA showed that the support for introducing virtual microscopy and autopsy presentations did not differ significantly from one another. When compared with any other method of teaching these two methods were each separately favoured significantly more (*p* < 0.05). In another subgroup analysis, which compared students who passed the pathology exam with students who had not yet passed it, the *t*-Test for independent variables also showed that students who already had completed pathology supported the development of seminars more than those who had not yet finished pathology (*p* = 0.04).

#### Correlation between student grades, lecture attendance and support for modernizing the curriculum

By means of a subgroup analysis the students who at the time of the survey had already finished all parts of the pathology course were investigated in more detail. Using this subgroup we were able to find that students who attended lectures did not have a significantly different grade to the students who did not attend lectures. The mean grade of the students who visited the lectures was 2.37 (SD 0.90) while that of those who did not visit the lectures was 2.34 (SD 0.96) (German grading system with 1 representing the highest grade).

Spearman’s correlation was used to investigate if there was a correlation between the students’ grade and their satisfaction with the current state in pathology teaching. Spearman’s rank correlation coefficient was calculated to be 0.24 suggesting a slightly positive correlation (*p* < 0.01). The same test of rank correlation was used to investigate the relationship between the students’ grades and whether the students wanted the pathology curriculum to be changed. This resulted in a negative correlation between grade and support of changing the curriculum i.e. the better the grade the less likely the students were to support changing the curriculum. Spearman’s rank correlation coefficient was −0.47 with statistical significance at *p* < 0.01 and showed moderate correlation.

## Discussion

Since the major contributions made to the field by Rudolph Virchow, the foremost cellular pathologist of the 19th century [8], the field has split into subspecialties and become more complex. It remains however that pathology is the study of the origin of and changes caused by disease. We found that students are willing to learn the basics of pathology which in our opinion should be the aim of current pathology teaching. The way such content is taught is important as it influences students’ views of the field and what they retain.

As a mixed method study the data presented consisted of a qualitative and quantitative part. The aim was to understand the students and their preferences. While the mixed method approach seemed appropriate to answer our questions, we are aware of some methodological limitations of our study. We sent LMU students to learn how the “modern” SOM teaching system in pathology works. However, we did not have SOM students study the “old fashioned” LMU teaching approach. While this would be a preferable and more comprehensive way of data collection it was not feasible in our setting, as no Australian students were visiting LMU. We nevertheless believe that even without this supplementary leg of the study our data allows for knowledge gain to improve pathology teaching.

As can be inferred from our quantitative data student satisfaction with an almost purely lecture-based curriculum is not very high with 79 % of students supporting the modernization of the curriculum (see Fig. 2). Using only lectures has been identified as an archaic model of teaching and faculties have realized this and pressed for a change [9, 10]. When in a lecture most students sit and try to listen but many end up experiencing “task-unrelated images and thoughts” [11]. In our study, with a large cohort of students, we were able to show that there was no statistically significant difference in the grades of students who attended lectures and those who didn’t. Subsequently this raises the question: “What’s the use of the lecture?” [12], as in the title of Donald Bligh’s 1998 book on the topic.

Why then do so many pathology curricula base their education mainly on lectures? In 2001 Kumar et al. published a survey suggesting that some 53 % of pathology teaching programs in US medical schools still used lectures as the main mode of teaching [5]. A possible explanation for the perseverance of lectures in pathology education is the fact that medical school class sizes have been increasing in the past decades [13, 14] making a “mass-lecture” a financially attractive teaching option. Donner and Bickley suggest that novel methods such as problem-based learning are only financially feasible with a class size below 60 [15]. With both LMU and SOM class sizes exceeding 400 students one can no longer assume that there is a teaching method more financially feasible than the lecture. One must also not forget that lectures do play an important role as an introduction into a field or course, and that there are learning types which benefit from lectures.

SOM employs the “tutorial” which is a form of teaching similar to problem-based learning. In the setting of such a high class size a large faculty (paid and unpaid) makes this possible which subsequently affects financial feasibility. It is thus clear that to be able to provide students with such courses one must have the ability to finance them or have in-kind support. This displays a key limitation of curriculum development, namely its link to faculty support (which can often be difficulty to gain) and the financial resources required to achieve positive changes.

Indeed education is not about financial feasibility but about the student’s success. In a study conducted in 2011, Arjun Singh was able to demonstrate the positive effect of the “patient-oriented problem-solving system” (POPS—a form of problem-based learning) on knowledge retention in his students [16]. Students who participated in POPS improved their quiz score by 439 % from their pre-course score, while those only receiving traditional lectures improved their score by a mere 204 %. After trialling POPS, 93 % of the students favoured this method. Julian Burton, who is a researcher in the field of pathology education, states that “pathology lends itself to problem-based learning” [17]. In the current study students showed support for an interactive teaching session which is referred to a seminar in Munich (tutorial at SOM). Students who had finished pathology courses were found to support this method even more than those who had not finished the course yet. This suggests that as one experiences traditional pathology teaching one develops the feeling that there is something important missing. One must however keep in mind the major time investment required of faculty for interactive teaching. The sessions include preparation, in class support of students and depending on the modality also evaluation and support of students after class. To implement such courses an involved and dedicated staff is required who might even need to invest time outside of the regular teaching time.

Further methods supported by students surveyed included virtual microscopy where students can digitally pan and zoom into pathological specimens. Fred Dee describes that by 2009 ca. 50 % of the pathology departments in the US were using virtual microscopy [18] with the main venue being student education. In Magdeburg, Germany the use of virtual techniques has even been expanded to macroscopic specimens which were digitalized and made available online to the students [19]. Nowadays it is highly appropriate for novel computer-based methods to be used as teaching resources.

The idea of accessing educational resources from afar has been addressed at SOM with lectures having been filmed and stored online for student use. LMU students voiced their support for the use of podcasts as shown in the data above, which ranks podcasts as the fourth most favourite mode of teaching with overall 75 % supporting and strikingly 41 % strongly supporting its use. In our study, the autopsy (87 %; 55 %) was ranked even higher than the podcast, with significantly more support (ANOVA data). This modality is one which was shunned at its beginnings [20] but which advanced to an important part of medical education and now has once again declined in use. An 18 state US study showed that medical students had experienced an average of one autopsy in their education, with 20 % of students never having even seen one at all [21]. In a qualitative context study of interviews performed in the UK in 2003, Julian Burton was able to find that the interviewed pathologists, physicians and surgeons believed that autopsies should play a role in medical education [22].

Motivations behind improving curricula can be student satisfaction and student grades. Our survey data suggests that the two are linked (Spearman’s rank correlation coefficient 0.24) and thus improving grades would increase satisfaction with the course. This hypothesis must be viewed critically as Kumar et al. present data that suggests changing curricular methods does not correlate with pathology testing performance [23, 24]. A subgroup analysis of the 10,159 students included in the described study looked specifically at students with low scores on standardized tests. This analysis found a correlation between their performance and changes in curricula [23], revealing that we can help these students with curricular changes. Regarding pathology course hours a further study was able to show that high course hours do not translate into better performance in the discipline [25]. As increasing teaching hours is a costly act, these are very convenient results for the pathology teaching community.

Working with a set amount of pathology teaching hours a good choice of teaching modality is important to be able to reach those students who actually need help learning. Our experience at SOM showed us that their form of problem-based learning and the use of computer-based methods as well as remote learning is a very attractive approach. This coincides with our survey results, which paint the picture of an ideal curriculum strongly resembling the program we found in Queensland.

## Conclusion

In 2006 Professor Paola Domizio of the University of London wrote that “the profile of pathology must once again be raised” and that “its loss in the modern curriculum must be corrected” [26]. With our data on what students want in pathology education we wish to motivate pathology departments around the globe to critically evaluate how the subject is taught at their school and whether their curriculum requires rejuvenation. The comparison of our survey results with the best practice model found at SOM showed that modernization is supported by students and that student surveys should be considered as an effective instrument to identify better teaching approaches for curriculum development. As LMU is not the only university utilizing traditional teaching methods our data will most certainly translate to further medical schools. We are hopeful that many schools will soon work on the modernization of their curricula in pathology and beyond to better support student-centred learning in the future.

## Abbreviations

ANOVA: Analysis of variance
IPLC: Integrated pathology learning centre (IPLC)
LMU: Ludwig Maximilian University, Munich
POPS: Patient-oriented problem-solving system
SD: Standard deviation
SOM: University of Queensland School of Medicine
